# The cancer driver genes *IDH1/2, JARID1C/ KDM5C*, and *UTX/ KDM6A*: crosstalk between histone demethylation and hypoxic reprogramming in cancer metabolism

**DOI:** 10.1038/s12276-019-0230-6

**Published:** 2019-06-20

**Authors:** Soojeong Chang, Sujin Yim, Hyunsung Park

**Affiliations:** Department of Life Science, University of Seoul, Seoul, 02504 Korea

**Keywords:** Epigenetics, Cancer

## Abstract

Recent studies on mutations in cancer genomes have distinguished driver mutations from passenger mutations, which occur as byproducts of cancer development. The cancer genome atlas (TCGA) project identified 299 genes and 24 pathways/biological processes that drive tumor progression (Cell 173: 371-385 e318, 2018). Of the 299 driver genes, 12 genes are involved in histones, histone methylation, and demethylation. Among these 12 genes, those encoding the histone demethylases JARID1C/KDM5C and UTX/KDM6A were identified as cancer driver genes. Furthermore, gain-of-function mutations in genes encoding metabolic enzymes, such as isocitrate dehydrogenases (IDH)1/2, drive tumor progression by producing an oncometabolite, D-2-hydroxyglutarate (D-2HG), which is a competitive inhibitor of α-ketoglutarate, O_2_-dependent dioxygenases such as Jumonji domain-containing histone demethylases, and DNA demethylases. Studies on oncometabolites suggest that histone demethylases mediate metabolic changes in chromatin structure. We have reviewed the most recent findings regarding cancer-specific metabolic reprogramming and the tumor-suppressive roles of JARID1C/KDM5C and UTX/KDM6A. We have also discussed mutations in other isoforms such as the JARID1A, 1B, 1D of KDM5 subfamilies and the JMJD3/KDM6B of KDM6 subfamilies, which play opposing roles in tumor progression as oncogenes or tumor suppressors depending on the cancer cell type.

## Cancer: Interplay between histone modification and tumor metabolism

Genes involved in the removal of methyl groups from histones associated with DNA can promote or suppress tumor growth depending on the metabolic status of the cancer cell. Hyunsung Park and colleagues at the University of Seoul, South Korea, review current knowledge of two genes encoding histone demethylases which have been identified by The Cancer Genome Atlas (TCGA) project as cancer driver genes. Because these demethylase enzymes rely on cellular metabolites to function, their effect is influenced by metabolic conditions in the tumor microenvironment such as low oxygen. The mechanisms through which changes in histone methylation affect the expression of genes involved in tumor progression remain unknown. Further understanding of how cancer metabolism affects the modification of histones will help guide the development of more effective cancer treatments.

## Introduction

**Fig. 1:**
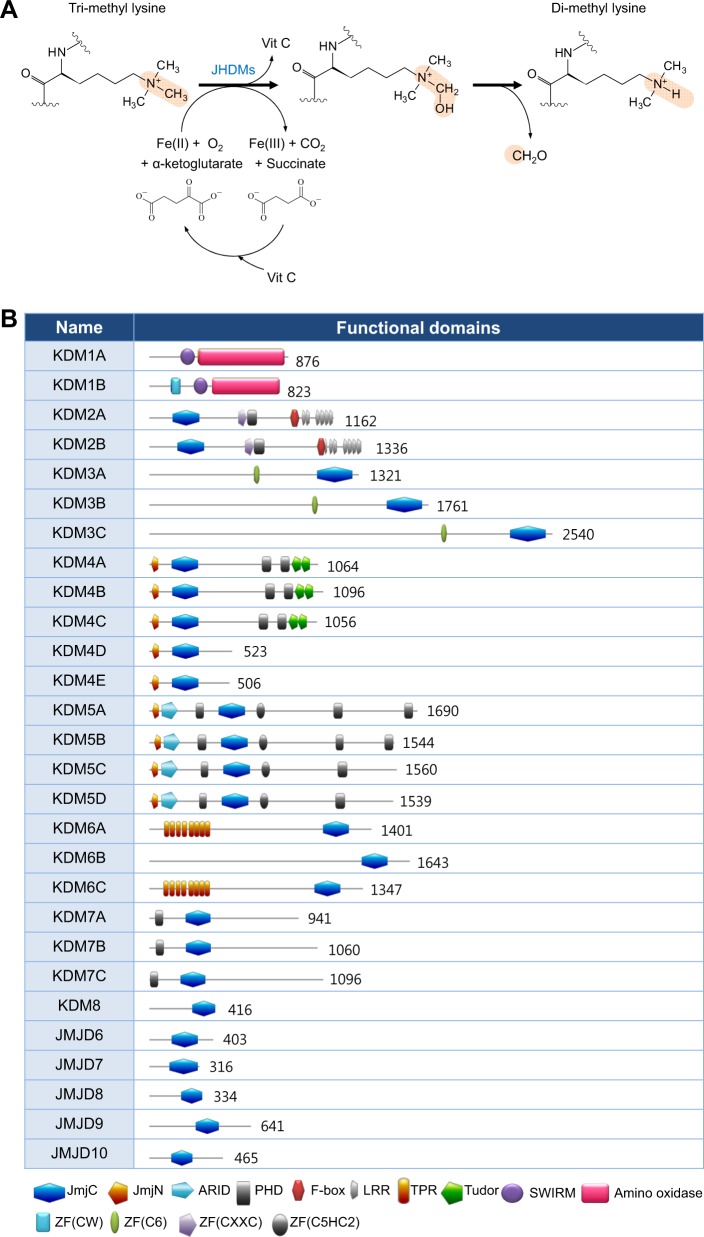
Functional domains and substrates of O_2_- and α-KG-dependent histone demethylases. **a** Substrates for O_2_- and α-KG-dependent histone demethylases. JHDMs (JmjC domain-containing histone demethylases) remove methyl (CH3-) groups from methylated lysines. JHDMs require O_2_, α-ketoglutarate, Fe(II), and vitamin C (vit C) for their catalytic activities and release CO_2_, succinate, and Fe(III). **b** Schematic illustration of the protein domains of histone demethylases. The domain architecture from the UniProt or NCBI gene (KDM1A, B) information was transformed using https://prosite.expasy.org/mydomains/. The numbers written in each functional domain column indicate the number of amino acids. ARID AT-rich interacting domain, amino oxidase amine oxidase domain, C5HC2 C5HC2 zinc-finger domain, CW CW-type zinc-finger domain, CXXC CXXC zinc-finger domain, C6 C6 zinc-finger domain, JMJC Jumonji C domain, LRR Leu-rich repeat domain, PHD plant homeodomain, SWIRM Swi3p, Rsc8p, and Moira domain, TPR tetratricopeptide domain, TUDOR Tudor domain

The three billion base pairs of DNA in the human genome contain genetic information that is inherited across generations; however, all genes are not accessible to the transcription machinery. Long DNA strands are packaged into chromatin structure, whose basic unit is the nucleosome, composed of the histone octamer (two copies each of H2A, H2B, H3, and H4) wrapped by two turns (147 bp) of DNA. These beads (nucleosomes)-on-a-string (DNA) structures are further organized into 30-nm-thick, compact heterochromatin structures. The three-dimensional structure of chromatin can be modulated by nucleosome positioning, modifications, and histone composition. Differences in chromatin structure can affect all events related to DNA, including transcription, replication, DNA repair, and splicing. Posttranslational modifications (PTMs) of histones critically affect interactions between histones and DNA and the recruitment of “reader” proteins, which recognize special histone modifications. For example, heterochromatin protein (HP)-1 specifically interacts with the trimethylated ninth lysine of histone 3 (H3K9me3) and triggers chromatin compaction via self-oligomerization. H3K9me3 and HP-1 are often detected in inaccessible heterochromatic regions. H3K27me3 also occurs in heterochromatic regions and is involved in transcriptional repression. In contrast, H3K4me3 occurs at the transcription start sites of active genes in euchromatic regions. Genome-wide analyses using RNA-seq and histone chromatin immunoprecipitation (ChIP)-seq elucidated the relationship between transcription and histone modification, which is more extensive than other molecular events on nucleosomes. Compact regions of the genome are less accessible for both the transcription and replication machineries^1^. Histone PTMs are determined by the balance between “writer” and “eraser” enzymes. These enzymes use cellular metabolites and coenzymes such as α-ketoglutarate (α-KG), S-adenosyl-methionine (SAM), flavin adenine dinucleotide (FAD), ATP, oxygen, Fe(II), acetyl-CoA, and vitamin C for their catalytic reactions^2,3^. For example, H3 lysine 4 (H3K4) can be methylated by MLL3/KMT2C or MLL4/KMT2D, members of the COMPASS family of histone H3K4 methyltransferases (writers), using SAM as the methyl donor, whereas H3K4me3 and H3K4me2 are demethylated by Jumonji domain-containing histone demethylases (erasers) (Table 1). The JARID1/KDM5 isoforms use oxygen, α-KG, vitamin C, and Fe(II) as cosubstrates (Fig. 1a).

**Table 1:** Cancer driver genes involved in epigenetics

Pathways involved in epigenetics	Driver genes	Tumor suppressor/oncogene prediction (by 20/20+^a^)	Approved name	Activity	Cancer type^b^	Other driver genes in this pathways
Histone modification	*KDM6A*	tsg	Lysine demethylase 6A, UTX	H3K27me2/3 demethylase	BLCA, HNSC, KIRP, LUSC, PAAD, PANCAN, PRAD	*PPP6C*
	*SETD2*	tsg	SET domain-containing 2	H3K36 methyl transferase	KIRC, KIRP, LGG, LUAD, MESO, PANCAN	
Chromatin histone modifiers	*KDM5C*	tsg	Lysine demethylase 5C, JARID1C	H3K4me2/3 demethylase	KIRC, PANCAN	*ARID5B*, *CREBBP*, *EP300*, *KANSL1*, *MEN1*, *NCOR1*, *NSD1*, *SIN3A*, *WHSC1*, *ZMYM3*
	*KMT2A*	tsg	Lysine methyltransferase 2A	H3K4 methyl transferase	PANCAN	
	*KMT2B*	tsg	Lysine methyltransferase 2B	H3K4 methyl transferase	PANCAN, UCEC	
	*KMT2C*	tsg	Lysine methyltransferase 2C	H3K4 methyl transferase	BLCA, BRCA, CESC, PANCAN, UCEC	
	*KMT2D*	tsg	Lysine methyltransferase 2D	H3K4 methyl transferase	BLCA, CESC, DLBC, ESCA, HNSC, LUSC, PANCAN, PRAD	
Chromatin (other)	*H3F3A*	Possible oncogene	H3 histone family member 3A, H3.3A		PANCAN	*AJUBA*, *ASXL1*, *ASXL2*, *ATF7IP*, *BCOR*, *CHD3*, *CHD4*, *CHD8*, *CTCF*, *NIPBL*, *NPM1*
	*H3F3C*	—	H3 histone family member 3C, H3.5		PANCAN	
	*HIST1H1E*	Possible oncogene	HIST1H1E, H1.4		DLBC	
		Possible tsg	HIST1H1E, H1.4		LIHC	
Metabolism	*IDH1*	Oncogene	Isocitrate dehydrogenase (NADP(+)) 1	NADP-dependent IDH, Cytosolic	CHOL, GBM, LAML, LGG, LIHC, PANCAN, PRAD, SKCM	—
	IDH2	Oncogene	Isocitrate dehydrogenase (NADP(+)) 2	NADP-dependent IDH, Mitochondrial	LAML, LGG, PANCAN	

Among the 299 driver genes mentioned by Bailey et al.^47^, only the epigenetics-related pathways have been sorted out

^a^20/20+: Classifies genes as an oncogene, tumor suppressor gene, or as a nondriver gene using Random Forests, http://2020plus.readthedocs.org

^b^BLCA (bladder urothelial carcinoma), BRCA (breast invasive carcinoma), CESC (cervical squamous cell carcinoma and endocervical adenocarcinoma), CHOL (cholangiocarcinoma), DLBC (lymphoid neoplasm diffuse large B-cell lymphoma), ESCA (esophageal carcinoma), GBM (glioblastoma multiforme), HNSC (head and neck squamous cell carcinoma), KIRC (kidney renal clear cell carcinoma), KIRP (kidney renal papillary cell carcinoma), LAML (acute myeloid leukemia), LGG (brain lower grade glioma), LIHC (liver hepatocellular carcinoma), LUAD (lung adenocarcinoma), LUSC (lung squamous cell carcinoma), MESO (mesothelioma), PAAD (pancreatic adenocarcinoma), PANCAN (Pan-cancer), PRAD (prostate adenocarcinoma), SKCM (skin cutaneous melanoma), THCA (thyroid carcinoma), UCEC (uterine corpus endometrial carcinoma)

More than 20 different Jumoni domain-containing histone demethylases have been identified (Fig. 1b), and their specific substrates and acronyms have been summarized in Table 2. Although these demethylases share common cosubstrates, their *K*_m_ values for each substrate vary. We have summarized the *K*_m_ values for O_2_ and α-KG for each histone demethylase isoform in Table 3. The O_2_ *K*_m_ values of the H3K9me2 and me1 demethylase JMJD1A/KDM3A are lower than those of other isoforms, such as JMJD2A; thus, the catalytic activity of JMJD1A persists even under severe hypoxia^4,5^. Therefore, the catalytic activities of histone demethylases are differentially regulated in response to changes in the concentrations of their cosubstrates. The sensitivities of each histone demethylase to oncometabolites such as D(R)-2-hydroxyglutarate (HG) and L(S)-2HG vary, suggesting that histone methylation can be differently altered in response to IDH mutations and the presence of oncometabolites (Table 3)^6^.

**Table 2:** O2- and α-KG-dependent histone demethylases

Symbol	Synonyms	Chromosome	Histone substrate selectivity^130^	Mutations in human cancer^a^
KDM1A	AOF2, KDM1, KIAA0601, BHC110, LSD1	1p36.12	H3K4me2/3, H3K9me1/2	UCEC (6.42%), READ (5.11%), BLCA (3.40%)
KDM1B	C6orf193, AOF1, FLJ34109, FLJ33898, dJ298J15.2, bA204B7.3, FLJ43328, LSD2	6p22.3	H3K4me1/2	UCEC (9.06%), STAD (4.77%), COAD (4.75%)
KDM2A	FBXL11, KIAA1004, FBL11, LILINA, DKFZP434M1735, FBL7, FLJ00115, CXXC8, JHDM1A	11q13.2	H3K36me1/2	UCEC (10.94%), CESC (5.54%), COAD (5.00%)
KDM2B	FBXL10, PCCX2, CXXC2, Fbl10, JHDM1B	12q24.31	H3K4me3, H3K36me1/2	UCEC (10.75%), COAD (8.50%), DLBC (8.11%)
KDM3A	JMJD1, JMJD1A, TSGA, KIAA0742, JHMD2A	2p11.2	H3K9me1/2	UCEC (6.98%), STAD (4.77%), BLCA (3.88%)
KDM3B	C5orf7, JMJD1B, KIAA1082, NET22	5q31.2	H3K9me1/2	UCEC (13.58%), SKCM (8.32%), COAD (6.50%)
KDM3C	TRIP8, DKFZp761F0118, KIAA1380, FLJ14374, JMJD1C	10q21.3	H3K9me1/2	UCEC (16.42%), STAD (8.86%), READ (7.30%)
KDM4A	JMJD2, JMJD2A, KIAA0677, JHDM3A, TDRD14A	1p34.2-p34.1	H3K9me1/2/3, H3K36me1/2/3, H1.4K26me1/2/3	UCEC (8.30%), COAD (5.75%), BLCA (3.40%)
KDM4B	JMJD2B, KIAA0876, TDRD14B	19p13.3	H3K9me1/2/3, H3K36me1/2/3, H1.4K26me1/2/3	UCEC (9.43%), COAD (7.25%), STAD (5.91%)
KDM4C	JMJD2C, GASC1, KIAA0780, TDRD14C	9p24.1	H3K9me1/2/3, H3K36me1/2/3, H1.4K26me1/2/3	UCEC (11.13%), STAD (4.09%), LUAD (4.06%)
KDM4D	JMJD2D, FLJ10251	11q21	H3K9me1/2/3, H3K36me1/2/3, H1.4K26me1/2/3	UCEC (6.98%), SKCM (3.20%), COAD (3.00%)
KDM4E	KDM4DL, JMJD2E, KDM5E	11q21	H3K9me1/2/3	UCEC (6.23%), LUAD (1.41%), CESC (1.38%)
KDM5A	RBBP2, JARID1A	12p13.33	H3K4me1/2/3	UCEC (18.87%), STAD (7.27%), SKCM (6.61%)
KDM5B	JARID1B, RBBP2H1A, PLU-1, CT31, PPP1R98	1q32.1	H3K4me1/2/3	UCEC (12.45%), STAD (6.59%), CESC (6.57%)
KDM5C	SMCX, JARID1C, MRX13, DXS1272E, XE169	Xp11.22	H3K4me1/2/3	UCEC (13.21%), STAD (6.36%), KIRC (6.25%)
KDM5D	HYA, HY, SMCY, JARID1D, KIAA0234	Yq11.223	H3K4me1/2/3	READ (2.19%), SKCM (1.71%), STAD (1.59%)
KDM6A	UTX	Xp11.3	H3K27me1/2/3	BLCA (29.37%), UCEC (13.21%), CESC (6.57%)
KDM6B	JMJD3, KIAA0346	17p13.1	H3K27me1/2/3	UCEC (11.32%), SKCM (8.10%), STAD (7.05%)
KDM6C	UTY, KDM6AL	Yq11.221	H3K27me1/2/3	BLCA (2.43%), ESCA (2.17%), READ (1.46%)
KDM7A	JHDM1D, KIAA1718	7q34	H3K9me1/2, H3K27me1/2, H4K20me1/2	UCEC (6.79%), SKCM (5.33%), COAD (5.00%)
KDM7B	ZNF422, JHDM1F, KIAA1111, PHF8	Xp11.22	H3K9me1/2, H4K20me1/2	UCEC (13.77%), SKCM (4.26%), CESC (3.81%)
KDM7C	KIAA0662, CENP-35, JHDM1E, PHF2	9q22.31	H3K9me1/2, H3K27me1/2, H4K20me3	UCEC (14.34%), COAD (6.25%), STAD (6.14%)
KDM8	JMJD5, FLJ13798	16p12.1	H3K36me2/3	UCEC (6.23%), COAD (3.00%), READ (2.92%)
JMJD6	PSR, PTDSR, PTDSR1	17q25.1	H3R2me1, H4R3me1	UCEC (4.91%), READ (3.65%0, DLBC (2.70%)
JMJD7	NA	15q15.1	NA	UCEC (2.64%), CESC (0.69%), SKCM (0.64%)
JMJD8	C16orf20	16p13.3	NA	DLBC (2.70%), UCEC (2.64%), UCS (1.75%)
JMJD9	RIOX1, C14orf169, MAPJD, NO66, ROX, URLC2, hsNO66	14q24.3	H3K4me2/3, H3K36me2/3	UCEC(5.66%), COAD (3.75%), STAD (2.95%)
JMJD10	RIOX2, MDIG, MINA, MINA53, NO52, ROX	3q11.2	H3K9me1/2	UCEC (8.30%), COAD (2.75%), STAD (2.5%)

^a^Identified in the Cancer Genome Atlas (TCGA, https://portal.gdc.cancer.gov/). AOF1/2, amine oxidase (flavin-containing) domain 1/2; BHC110, BRAF35-HDAC complex protein; CENP-35, centromere protein 35; CT31, cancer/testis antigen 31; CXXC2/8, CXXC-type zinc finger protein 2/8; Fbl7/10, F-box protein 7/10; FBXL10/11, F-box and leucine-rich repeat protein 10/11; GASC1, gene amplified in squamous cell carcinoma 1 protein; HYA, histocompatibility Y antigen; JARID, Jumonji/AT-rich interaction domain-containing protein; JHDM, Jumonji C domain-containing histone demethylase; JMJD, Jumonji domain-containing; KDM, lysine-specific demethylase; LRR, leucine-rich repeat; LSD1/2, lysine-specific histone demethylase 1/2; MAPJD, MYC-associated protein with JmjC domain; MDIG, mineral dust-induced gene protein; MINA(52/53), MYC-induced nuclear antigen (52/53); NO52/66, nucleolar protein 52/66; PHF2/8, plant homeobox domain finger protein 2/8; PCCX2, protein-containing CXXC domain 2; PPP1R98, protein phosphatase 1, regulatory subunit 98; PSR, phosphatidylserine receptor; PTDSR, Phosphatidylserine receptor; RBBP2, retinoblastoma binding protein 2; RIOX1/2, ribosomal oxygenase 1/2; ROX, ribosomal oxygenase; SMCX/Y, protein SmcX/Y; TDRD14A/B/C, Tudor domain-containing 14A/B/C; TPR, tetratricopeptide repeat; TSGA, testis-specific protein A; URLC2, upregulated in lung cancer 2; UTX/Y, ubiquitously transcribed tetratricopeptide repeat, X/Y chromosome; XE169, Protein Xe169

**Table 3:** *K*_m_ values for the substrates and IC50 values for oncometabolites of KDMs

Enzyme	KM (α-KG)/μM^a^	KM (O_2_)/μM^a^	KM (Fe^2+^)/μM^a^	IC50 (D-2HG)/μM^b^	IC50 (L-2HG)/μM^b^
KDM2A	NA	NA	NA	106 ± 22^26^	48 ± 15^26^
KDM4A	10 ± 1^131^ ~ 15 ± 9^6^	57 ± 10^131^	<0.1^6^	2.1^132^ ~ 24 ± 2^26^,160 ± 10^6^	26 ± 3^26^,290 ± 20^6^
KDM4B	6 ± 3^6^	NA	<0.1^6^	150 ± 30^6^	450 ± 130^6^
KDM4C	12 ± 2^131^	158 ± 13^131^	NA	79 ± 7^26, 133^	97 ± 24^26, 28^
KDM4D	NA	NA	NA	Inhibition^4^	Inhibition^4^
KDM4E	21 ± 2^131^	197 ± 16^131^	NA	NA	NA
KDM5B	10 ± 2^6^	NA	<0.1^6^	10870 ± 1850; Ki^27^, 3600 ± 1400^6^	628 ± 36; Ki^27^, 3600 ± 1400^6^
KDM5C	5.4 ± 0.5^134^	NA	NA	NA	NA
KDM6A	8 ± 4^6^ ~10 ± 1.3^135^	NA	<0.1^6^	180 ± 30^6^	180 ± 30^6^
KDM6B	8.2 ± 1.0^135^ ~50 ± 20^6^	NA	6 ± 2^6^	350 ± 100^6^	350 ± 100^6^
KDM6C	5.1 ± 1.4^135^	NA	NA	NA	NA
TET1	55 ± 20^136^	30 ± 10^136^	4.8 ± 4^136^	Inhibition^27^	Inhibition^27^
TET2	60 ± 15^136^	30 ± 3^136^	3.6 ± 3^136^	5000^4^, Inhibition^47^	1600^4^, Inhibition^27^
FTO	NA	NA	NA	Inhibition^137^	Inhibition^138^
FIH	25 ± 3^139^	90−150^140^	0.5 ± 0.2^139^	1000^4^ ~ 1500 ± 400^26^	189 ± 34^26^ ~ 300^4^
PHD1	1^141^	NA	NA	>50,000^141^ Activation (210 ± 30; *K*_m_^4^)	625 ± 100; Ki^141^
PHD2	60^142^, 2 ± 0.4^141^	250^142^	0.03 ± 0.004^142^	>50,000^141^, 7300 ± 3300^26^ Activation (300 ± 40; *K*_m_)^141^	1150 ± 130; Ki^141^, 419 ± 150^26^
PHD3	60^142^, 12^141^	NA	NA	>50,000^141^	90 ± 20; Ki^141^
P4HA1/2, PLOD1/3	20^143^	40^143^	2^143^	∼2000^141^	NA
ALKBH2	NA	NA	NA	424 ± 77^26^ ∼ 500^144^	150 ± 20^26^
ALKBH3	NA	NA	NA	500^144^	NA
ATP5B	NA	NA	NA	Inhibition^145^	Inhibition^145^
BBOX1	NA	NA	NA	13,200 ± 1100^26^	142 ± 30^26^

*ALKBH2/3* AlkB homolog 2/3, *ATP5B* ATP synthase β subunit, *BBOX1* γ-butyrobetaine hydroxylase 1, *FIH* factor inhibiting-hypoxia-inducible factor, *HIF* hypoxia-inducible factor, *2OG* 2-oxoglutarate, *PHD1/2/3* prolyl hydroxylase domain-containing protein 1/2/3, *PLOD1/3* collagen lysine hydroxylases, *P4HA1/2* collagen prolyl hydroxylases, *R-2HG* R-enantiomer of 2-hydroxyglutarate, *IC50* half-maximal inhibitory concentration, Ki inhibitory constant

^a^The *K*_m_ values of KDM4A/4C/4E/6A/6B/6C, TET1/2, FIH, PHD1/2/3, P4HA1/2, PLOD1/3, ALKDH2/3 for their substrates α-KG, oxygen, and Fe^2+^ were determined. The values are mean (±SD)

^b^Inhibition of 2OG-oxygenases by R- and S-2HG for KDM2A, KDM4A/4C/4D/5B, TET1/2, FTO, FIH, PHD1/2/3, P4HA1/2, PLOD1/3, ALKBH2/3, ATP5B, and BBOX1. The values are mean (±SD

**Fig. 2:**
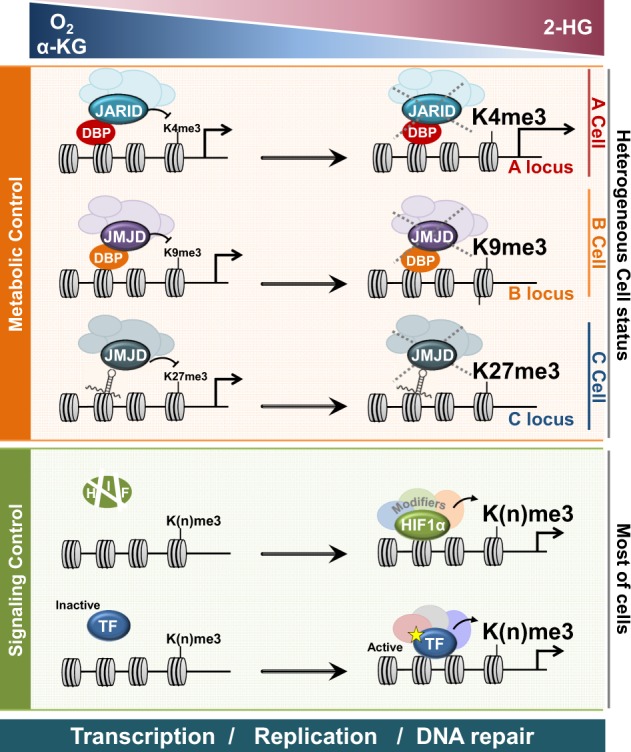
Schematic showing signal control vs. metabolic control. Metabolic control and signaling control interpret how the levels of O_2_, α-ketoglutarate (α-KG), and 2-hydroxyglutarate (2-HG) modulate gene expression by altering histone methylation. DNA-bound proteins (DBP) or DNA-bound long noncoding RNAs recruit histone demethylases (KDM), such as JARID and JMJD. In low oxygen or α-KG concentrations and a 2-HG high concentration, the activities of JARID or JMJD are suppressed at different loci for each cell, resulting in a high methylation level on the corresponding loci. Transcription factors (TF) such as hypoxia-inducible factor (HIF) are activated under low oxygen conditions or high 2-HG conditions. Activated TFs recruit histone methyltransferase (HMT), which forms a complex with KDM. In most cells, although KDM and HMT are recruited together, the histone methylation level was upregulated by HMT, as the KDM activity is suppressed under hypoxic or high 2-HG conditions. Dynamic histone modification can regulate transcription, replication, and DNA repair

Although histones are the most abundant molecules in the nucleus, each is fixed at different positions and is hence immobile inside the nucleus. Each histone is unique, as they are wrapped by different DNA sequences. Histone modification enzymes, both writers and erasers, cannot recognize specific DNA sequences in their targets (except for JARID1) but can be recruited in the vicinity of specific histones via interactions with transcription factors (TF) or long noncoding RNAs (lncRNA), which can recognize and interact with specific DNA sequences. Therefore, only a certain subset of histones can be modified when the enzymes are sufficiently close to their substrate for a chemical reaction to occur. In the “signal control” mechanism, certain signals select specific histone substrates and alter their methylation status by activating specific TFs or lncRNAs, which recognize DNA sequences to recruit specific histone methyltransferases and/or demethylases in the vicinity of the selected histones (Fig. 2). In contrast, “metabolic control” mechanisms instantly change histone methylation without activation of additional TFs. Changes in the metabolic substrates or inhibitors can instantly alter the activities of enzymes that are already recruited on specific regions of the genome, resulting in altered levels of histone methylation. Therefore, metabolic control of histone modification depends on (i) the availability of substrates and inhibitors and (ii) the genomic regions where the writers and erasers already reside. As the designated histone molecules where the writers and erasers reside are cell-specific, the effects of metabolic control of histone modification vary within cells, thereby increasing cellular heterogeneity (Fig. 2).

Hypoxic tumor microenvironments have been considered the main cause for increased chemo- and radiotherapy resistance. Recent studies revealed that hypoxic conditions limit the effective concentration of α-KG and increase the 2-HG level via metabolic reprogramming. Although several studies showed that hypoxia itself, hypoxia-induced metabolic reprogramming, and *IDH* mutations increase the total abundance of methylated histones in different cancer cells, the mechanism through which the increased level of histone methylation is related to cellular heterogeneity, cancer resistance, and progression remains unknown. This review introduces recent observations regarding (i) metabolic reprogramming of the α-KG balance by *IDH* mutation and hypoxia in cancers, (ii) the tumor-suppressive functions of the cancer driver genes *JARID1C/KDM5C* and *UTX/KDM6A*, and (iii) mutations in genes encoding other isoforms in various cancers, namely, JARID1A, 1B, 1D, and JMJD3/KDM6B of the KDM5 and KDM6 subfamilies.

## Hypoxic reprogramming of the α-KG balance in cancer metabolism

### Production of an oncometabolite, D(R)-2HG

**Fig. 3:**
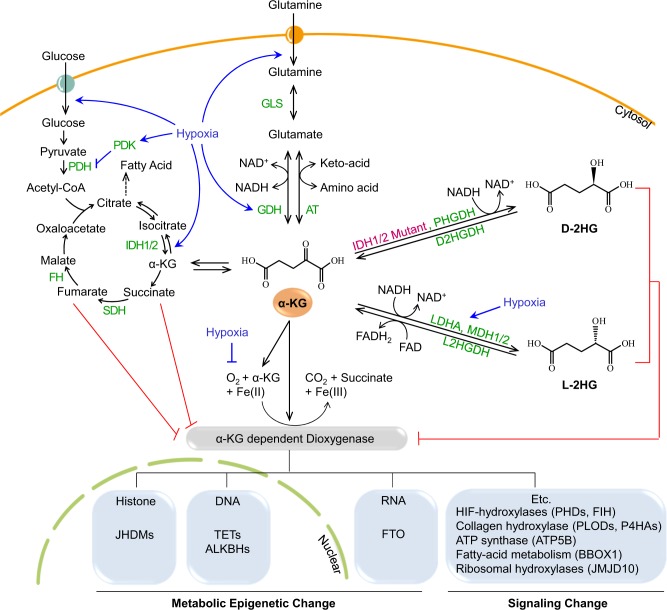
Metabolic reactions that alter the effective α-KG concentration. Glucose and glutamine are transported inside cells using individual transporters (circles with arrows). Glucose is converted to pyruvate via glycolysis, which is then converted into acetyl CoA by PDH and used in the TCA cycle. Intermediates in the TCA cycle, from citrate to oxaloacetate, are converted by individual enzymes. IDH1 and IDH2 are localized in the cytosol and mitochondria, respectively. Both enzymes convert isocitrate to α-KG. Glutamine is converted to glutamate by GLS, which can be converted to α-KG by GDH or AT. α-KG is used as a substrate by α-KG-dependent dioxygenase together with oxygen and iron ions. The types of α-KG-dependent dioxygenases include enzymes that induce metabolic epigenetic changes in histones, DNA, and RNA, and enzymes that induce changes in signaling. As these enzymes are α-KG- and O_2_-dependent, they are inhibited via deprivation of these factors. Regarding α-KG, (i) mutated IDH1/2 and PHGDH produce D-2HG, and (ii) LDHA and MDH1/2, whose levels are increased under hypoxic conditions, produce L-2HG. Both types of 2-HG compete with α-KG to inhibit α-KG-dependent dioxygenases. Both D-2HG and L-2HG can be converted to α-KG by D2HGDH and L2HGDH, respectively. Under hypoxic conditions, the activity of α-KG-dependent dioxygenases is inhibited via the regulation of glucose and glutamine pathways as well as by the O_2_ concentration. Hypoxia increases glycolysis by upregulating glucose transporters and enzymes related to glycolysis. Hypoxia-inducible factor 1 alpha (HIF1α) activates PDK, an enzyme that inactivates PDH (which converts pyruvate to acetyl-CoA); therefore, hypoxia inhibits the entry of pyruvate into the TCA cycle. Glutamine uptake and GDH (the enzyme that converts glutamate to α-KG) are upregulated under hypoxic conditions. Glutamine is converted to α-KG to provide intermediates for the TCA cycle. Details are provided in the text and the enzymes are shown in green. AT aminotransferases, D2HGDH D-2HG dehydrogenase, FH fumarate hydratase, FTO fat mass and obesity-associated protein, GDH glutamate dehydrogenases, GLS glutaminase, IDH1/2 isocitrate dehydrogenases 1/2, JHDM JmjC domain-containing histone demethylases, L2HGDH L-2HG dehydrogenase, LDHA lactate dehydrogenase A, MDH 1/2 malate dehydrogenase 1/2, PDH pyruvate dehydrogenase, PDK1 pyruvate dehydrogenase kinase 1, PHGDH D-3-phosphoglycerate dehydrogenase, SDH succinate dehydrogenase, TET ten-eleven translocation, TCA tricarboxylic acid

Supra-physiological concentrations of the D(R) form of 2-hydroxyoxoglutartate (D-2HG or R-2HG), a stereo-specific isoform of the natural and endogenous L-2HG (or S-2HG) molecule, was detected in the sera of patients with acute myeloid leukemia (AML) and glioma^7,8^. Cancer genomic analyses have identified that an abnormal increase in the D-2HG level is associated with mutations in either *IDH1* or *IDH*2. Wild-type IDH1 and IDH2 catalyze the oxidative decarboxylation of isocitrate to generate α-KG and CO_2_ (Fig. 3). IDH1 localizes to the peroxisomes and cytosol, whereas IDH2 localizes to the mitochondria. In several tumors, including glioma, AML, chondrosarcoma, intrahepatic cholangiocarcinoma, and thyroid carcinoma (Table 1), missense mutations of arginine residues in the active sites of both IDH1 and IDH2 (IDH1^R123H^, IDH2^R140Q^, or IDH2^R172K^) result in new activities that further convert α-KG to D-2HG^9–17^. The observation that D-2HG is sufficient to promote transformation of hematopoietic cells suggested that R-2HG is an oncometabolite^18^. Specific inhibitors of these IDH mutants have been developed and some are in clinical trials^19,20^. Nonetheless, the molecular mechanisms through which D-2HG promotes tumorigenesis remain poorly understood. Since D-2HG is a competitive inhibitor of α-KG-dependent dioxygenases, such as histone-, DNA- and RNA demethylases, D-2HG may induce dysregulation of histone, DNA, and RNA methylation in various cancer cell lines. In addition to mutated IDHs, D-3-phosphoglycerate dehydrogenase (PHGDH) also catalyzes the reduction of α-KG to D-2HG using NADH (Fig. 3)^21^. Accordingly, PHGDH is frequently amplified in breast cancer, and a subset of breast malignancies have been shown to accumulate 2-HG^22,23^. Under normal conditions, the D-2HG level remains low because of the catalytic activity of endogenous D-2HG dehydrogenase (D2HGDH), which catalyzes the conversion of D-2HG to α-KG; however, mutant IDHs produce excess D-2HG, which accumulates in patients with glioma and AML^18,24,25^.

### Excess endogenous L(S)-2HG in hypoxic cells

Similar to D-2HG (or R-2HG), excessive L-2HG (or S-2HG), an enantiomer of D-2HG, can also inhibit numerous α-KG-dependent enzymes^26,27^. Recent studies showed that hypoxia dramatically induces L-2HG production in both normal and malignant cells^28,29^. Under hypoxic or acidic conditions, L-2HG production was increased via promiscuous catalytic activities of lactate dehydrogenase A (LDHA) and malate dehydrogenase (MDH1 and 2) (Fig. 3). Purified LDH and MDH enzymes stereo-specifically reduced α-KG to L-2HG using NADH. Acidic conditions increased the levels of the protonated α-KG form, which more stably binds to the catalytic region of LDH-A, thereby increasing L-2HG production^30^. Hypoxic condition in the core region of many solid tumors enhanced L-2HG production via HIF-1α-dependent induction of LDH-A expression. Similar to D-2HG, L-2HG inhibits the Jumonji family histone lysine demethylase KDM4C, resulting in aberrant accumulation of trimethylated histone 3 lysine 9 (H3K9me3)^28^.

### D- and L-2HG accumulation due to defects in 2-HG dehydrogenases

L-2HG is oxidized and converted to α-KG by L-2HG dehydrogenase in the mitochondria. Similarly, D-2HG is also oxidized by D2HGDH. These 2-HG oxidation reactions are coupled with the reduction of FAD or NAD+^31^. Oxidation of 2-HG increases the NADH and FADH levels in cells; thus, 2-HG may act as a reservoir of reducing equivalents^32–34^. Consistent with this idea, the NADH/NAD ratio increases when mitochondrial respiration is impaired; limited 2-HG oxidation in hematopoietic stem cells increases the levels of 2-HG, which is accompanied by inhibition of DNA and histone demethylation, leading to increased hypermethylation of both DNA and histones^34^. Homozygous germline loss-of-function mutations in *D2HGDH* or L-2HG dehydrogenase (*L2HGDH)* increase the D-2HG or L-2HG levels, respectively, in both urine and blood^35^. Systemic L-2HG elevations arising from inherited *L2HGDH* mutations have been associated with brain tumors^36^. *L2hgdh* knockout mice display an increased L-2HG level in the brain with progressive leukoencephalopathy and neurodegeneration^37^.

### Glutamine deprivation and reduction of a-KG in cancer

Hypoxic environments in solid tumors reduce pyruvate production by inducing a less active M2 form of pyruvate kinase (PKM2) in an HIF-1α-dependent manner, such that hypoxia enhances glycolysis but limits oxidative phosphorylation^38–40^. Under hypoxic conditions, glutamine (the most abundant amino acid in blood) is used as the major precursor that can be converted into intermediates of the tricarboxylic acid cycle to support cancer cell survival and proliferation by generating nucleotides, amino acids, and fatty acids. Glutamine is transported into the cytoplasm by transporters such as solute carrier family 1 (neutral amino acid transporter) member 5 (SLC1A5), followed by conversion to glutamate by glutaminase^41^. Glutamate can be converted to α-KG either by glutamate dehydrogenases, or by aminotransferases^42^. Therefore, in hypoxic tumor environments, α-KG production depends on glutamine supplied by the blood. The increased glutamine catabolism in tumors may deplete the local supply, leading to glutamine deprivation. This possibility is supported by in vivo findings that the glutamine level decreases to almost undetectable levels in numerous tumors, including hepatomas and sarcomas^43–45^. A recent study using metabolomic analysis comparing paired pancreatic tumor patient samples with benign adjacent tissue specimens revealed that glutamine is one of the most strongly depleted metabolites in tumors^44^. Glutamine is further depleted in the hypoxic core regions of tumors due to poor blood supply and increased consumption by multiple anaerobic metabolic processes^46^. Using patient-derived melanoma, Pan et al. showed that glutamine deficiency also contributed to drug resistance and tumor heterogeneity^45^. They showed that glutamine depletion increased the abundance of methylated histone via α-KG reduction, a substrate of KDMs. Knockdown of *KDM6B/JMJD3* (H3K27me3 demethylase) reproduced the effects of low glutamine, suggesting that O_2_- and α-KG-dependent histone demethylases mediate signals from tumor microenvironments and metabolic status to chromatin.

Several studies showed that hypoxia in tumors contributes to inhibition of histone demethylases via multiple processes: (i) by limiting their substrate O_2_, and (ii) by reprogramming anaerobic metabolism to deplete α-KG and increase 2-HG, an inhibitor of α-KG. Furthermore, mutations in *IDH1* and *2* in several cancers result in high D-2HG levels. Many studies have shown that hypoxia and the oncometabolite increase the total amount of methylated histones in various cancer cells. However, there are many unsolved questions: (i) does this metabolic control of histone methylation vary with single cell status? (ii) Is this metabolic regulation related to tumor-suppressive functions of the cancer driver genes *JARID1C/KDM5C* and *UTX/KDM6A?* (iii) What is the molecular mechanism through which changes in histone methylation influence tumor progression? Several studies estimated the *K*_m_ values for either O_2_ and α-KG and the IC_50_ for 2-HG of several histone demethylases and other dioxygenases, demonstrating that each dioxygenase has different sensitivities for substrates and inhibitors (Table 3). Next, we will discuss the tumor-suppressive and oncogenic functions of the KDM5 and KDM6 subfamilies, among which the JARID1C/KDM5C and UTX/KDM6A isoforms have been identified as cancer drivers via TCGA analyses (Table 1)^47^.

## *JARID1C/KDM5C*, a cancer driver gene, and other isoforms of the KDM5 (H3K4me3, me2, and me1 demethylases) subfamily

TCGA predicted JARID1C/KDM5C as a tumor suppressor, mutations in which drive cancer progression (Table 1). JARID1C/KDM5C is one of the four different isoforms of the JARID1/KDM5 subfamily, which catalyzes demethylation of H3K4me3, me2, and me1 (Tables 1 and 2). Similar to other histone demethylases, JARID1 isoforms have different names and cell type-specific expression patterns. Several studies have revealed the diverse roles of JARID 1A, 1B, and 1D both as tumor suppressors and oncogenes depending on the type of cancer cells. JARID1 proteins contain multiple conserved domains (Fig. 1b). Their catalytic domains are located in two cores of the JmjN and JmjC domains, which are separated by two different domains, namely, an AT-rich interacting domain (ARID) and a plant homeodomain 1 (PHD1) finger^48^. The interaction between the JmjN and JmjC domains causes a conformational change in the catalytic core of JmjC, thereby activating the enzyme. The ARID domain in JARID1B recognizes the GCACA/C consensus motif, whereas the PHD1 domain binds only unmethylated H3K4; thus, JARID1B persists after demethylation and is involved in repressing target genes^49–51^. This function of PHD1 is unique for JARID1B but not for its other isoforms. Studies on yeast Jhd2 (a JARID1 homolog) showed that the C5HC2 domains and the other C-terminal PHD domain are dispensable for its catalytic activity, suggesting that the C-terminal domains of JARIDs participate in diverse interactions with other proteins to cell type-specific and nonenzymatic functions^52,53^.

### JARID1C/KDM5C as a tumor suppressor

*JARID1C* was identified as an X-linked mental retardation-related gene that escapes X inactivation during embryogenesis. Thus, females harbor two active copies of *JARID1C*, whereas males possess a single copy^54^. Loss of *JARID1C* in males contributes to sex bias phenotypes^55,56^. Mutations in *JARID1C* are associated with short stature, hyperreflexia, and autism^57,58^. Mutations in *JARID1C* have been identified in many cancers, such as clear cell renal cell carcinoma (ccRCC), pancreatic cancer, and human papillomavirus (HPV)-associated cancer (Table 1)^59–63^. In ccRCC, von Hippel Lindau (VHL), a tumor suppressor, is dominantly inactivated. As VHL is an HIF-α subunit-specific E3 ubiquitin ligase, HIF-α is constitutively activated in VHL-inactive ccRCC. Whole-exome sequencing analyses of ccRCC revealed that in addition to *VHL*, *JARID1C/KDM5C*, *UTX/KDM6A*, and *SETD2* (H3K36 methyltransferase) were new cancer genes in ccRCC^61^. In 786-O *VHL−/−* ccRCC cells, *JARID1C* knockdown significantly enhanced tumor growth in a xenograft mouse model, showing that *JARID1C* is a tumor suppressor and that its inactivating mutations in ccRCC promote tumors^60^. Whole-exome sequencing of human pancreatic cancers identified truncating insertions and deletion mutations in *JARID1C*^63^. Genome-wide siRNA screening has identified SMCX (another name of JARID1C) as an E2-dependent regulator of the HPV oncogenes E6 and E7^62^. The HPV protein E2 represses the expression of oncogenic E6 and E7 genes by inactivating p53 and Rb, respectively. JARID1C was shown to repress E6 and E7 by interacting with E2. *JARID1C* knockdown increased the expression levels of the E6 and E7 oncogenes, suggesting that JARID1C can function as a tumor suppressor in HPV-associated cancers.

The molecular mechanism through which JARID1C suppresses tumor growth remains unclear. One explanation is that JARID1C represses important oncogenic target genes by reducing the active H3K4me3 marks at several promoters, where it is recruited via interactions with DNA-binding proteins^64^; furthermore, JARID1C is recruited to the promoters of *IGFBP1, DNAJC12, COL6A1*, and *GDF15* by interacting with HIF in 786-O *VHL−/−* ccRCC cells^60^, and at the long control region promoter of the viral E6 and E7 genes via interaction with E2^62^. Rondinelli et al. suggested that in addition to transcriptional regulation, the H3K4me3 demethylase activity of JARID1C is also associated with heterochromatin assembly and genomic instability after replication in ccRCC cells^65^. JARID1C binds to heterochromatic regions where transcription is tightly suppressed. Demethylation of H3K4me3 by JARID1C is required for Suv39H1-mediated methylation of H3K9 for postreplication heterochromatin reconstruction. In ccRCC cells, JARID1C inactivation failed to reduce H3K4me3 at heterochromatic regions, preventing Suv38H1 H3K9 methyl transferase and HP1a from binding to heterochromatin. Under this condition, heterochromatin assembly is hindered, leading to unrestrained expression of aberrant noncoding RNAs, which trigger genomic rearrangement. Therefore, JARID1C prevents tumor progression by guarding genomic stability via maintenance of heterochromatic regions. JARID1C is also required for proper DNA replication by removing tri-methyl groups from H3K4 at replication origins, which drives origin binding by the preinitiation proteins CDC45 and PCNA^66^. Whether promotion of DNA replication is related to the tumor suppression function of JARID1C is unclear.

### Other KDM5 isoforms, JARID1A, 1B, and 1D, as tumor suppressors

JARID1A and 1B are not cancer drivers; however, they are the most intensively studied isoforms. Several studies provided evidence that they are involved in tumor suppression. JARID1A/KDM5A/RBP2/RBBP2 was identified as an Rb-interacting protein, while JARID1B/KDM5B/PLU-1 was identified as a gene that is upregulated by overexpression of c-ErbB2^67,68^. The H3K4 demethylation activities of both JARID1A and 1B mediate Rb’s function of repressing E2F target genes, suggesting that JARID1A and 1B may be involved in tumor suppression by enhancing cellular senescence^69^. Consistent with this idea, Nijwening et al. demonstrated that JARID1B depletion led to phenotypes similar to those observed after Rb1 knockdown and that JARID1B associates with E2F target genes during cellular senescence^70^. The genome-wide epigenetic state of leukemic stem cells in MLL-associated leukemia revealed that leukemic stem cells maintained a high level of H3K4me3 and a low level of H3K79me2. JARID1B negatively regulates self-renewal and the oncogenic potential of leukemic stem cells by reducing H3K4me3 abundance^71^. A genome-wide transcriptional analysis revealed that the JARID1B-LSD1-NuRD1 complex targeted the cell migration- and angiogenesis-related CCL14 chemokine pathway^52^. Experiments using MDA-MB-231 and MCF7 breast cancer cells showed that JARID1B suppressed cell invasion and angiogenesis by repressing CCL14 expression, suggesting that JARID1B suppresses tumor progression. JARID1D was originally reported as a minor histocompatibility antigen on the Y chromosome^72^. JARID1D was considered a tumor suppressor because of its downregulation, mutation, or loss in prostate cancer and ccRCC^73,74^. JARID1D directly interacts with the androgen receptor (AR) to attenuate the transcriptional activation of AR target genes in hormone-sensitive prostate cancers, whereas JARID1D expression is downregulated during the progression to hormone-resistant tumors.

These diverse roles for the JARID1 isoforms in multiple cancer types highlight the roles of JARID1A, 1B, and 1D as tumor suppressers; however, unlike JARID1C, the functions of JARID1A, 1B, and 1D depend on the cellular context, which underscores their potential as cancer biomarkers and drug targets. In contrast, several studies showed that JARID1A and 1B are involved in tumor progression rather than in suppression.

### Oncogenic function of JARID1A and 1B

*JARID1A* is significantly amplified and overexpressed in various human tumors, including breast cancer and head and neck squamous cell carcinoma^75,76^. Unlike *JARID1B*, *JARID1A* knockdown altered H3K4 methylation, resulting in inhibition of proliferation and reduced drug resistance, which is suggestive of the oncogenic roles of JARID1A in breast cancer^75^. Wang et al. (2009) reported that fusion of the C-terminal PHD domain of JARID1A/PHF23 to nucleoporin-98 generated potent oncoproteins in human leukemia. In the NUP98-PHD fusion protein, the ability of the PHD domain, which recognizes the H3K4me3/2 mark, was essential for leukemogenesis^77^. Lin et al. showed that loss of JARID1A/RBP2 promotes senescence and inhibits proliferation in a histone demethylase activity- and Rb-dependent manner in mouse embryonic fibroblasts (MEFs). Furthermore, genetic ablation of *JARID1A/RBP2* decreased tumor formation in the pancreatic islets of *Men1* (a tumor suppressor)-defective mice and in the pituitary glands of *Rb(+/−)* mice, thereby improving the survival of these mice. These results suggested that JARID1A activates tumorigenesis^78^. Similarly, *JARID1A* depletion inhibits proliferation, migration, invasion, and metastasis of lung cancer, suggesting oncogenic roles of JARID1A in lung tumorigenesis and progression^79^.

Roesch et al. have recently demonstrated that even within highly proliferative melanomas, there is a slow-cycling cell subpopulation with a doubling time of >4 weeks^80,81^. They observed that the expression level of the RBP2-homolog 1/JARID1B/KDM5B/PLU-1 is particularly high in the slow-cycling cell subpopulation. Melanomas are highly heterogeneous tumors, but the biological significance of their different subpopulations is not clear. The same authors characterized the slow-cycling subpopulation by isolating JARID1B^high^ melanoma cells. *JARID1B* knockdown leads to an initial acceleration of tumor growth, followed by exhaustion. The JARID1B^high^ subpopulation has cancer stem cell-like molecular and functional traits, which are essential for continuous tumor growth. The JARID1B^high^ slow-cycling melanoma cells were characterized by high expression levels of enzymes related to mitochondrial bioenergetics and increased drug resistance, suggesting that JARID1B contributes to the maintenance of cancer stem cell traits. Although the H3K4me3 demethylation activity of JARID1B is required for maintaining cancer stem cell traits, the mechanisms through which the JARID1 enzymatic activity is involved in the cell cycle, drug tolerance, and oxidative metabolism are unclear^82,83^. Similar to the heterogenic cell populations in melanoma, other types of cancers, such as head and neck cancers, also contain slow-cycling cell populations that show cancer stem cell traits and drug resistance^84^. Sharma et al. also isolated a drug-tolerant cancer cell population with cancer stem cell properties from an *EGFR*-mutant non-small cell lung carcinoma (NSCLC)-derived cell line (PC9). They observed that JARID1A is required for maintaining the drug-tolerant cancer cell population^85^. These studies showed that JARID1A and 1B contribute to intratumor heterogeneity and cancer stem cell traits, which are required for tumor progression.

## *UTX/KDM6A*, a cancer driver gene, and other isoforms in the KDM6 (H3K27me3, me2, and me1 demethylase) subfamily

Two functional isoforms, ubiquitously transcribed tetratricopeptide repeats on the X chromosome (UTX)/KDM6A and JMJD3/KDM6B, have been identified as H3K27me3-, me2- and, me1-specific histone demethylases. Similar to *JARID1C*, *UTX* has been identified as an X-linked gene that escapes X chromosome inactivation and is ubiquitously expressed. This escape from X-inactivation contributes to sex bias in many tumors, as a single mutation in *UTX* is sufficient for its loss of function in males but not in females^56^. The UTX paralog, Y chromosome-linked UTY, is inactive due to a mutation in the JmjC domain^86,87^. Both UTX and JMJD3 have C-terminal JmjC domains, which possess catalytic activities. UTX/KDM6A, but not JMJD3/KDM6B, harbors N-terminal six tetratricopeptide repeat (TPR) domains that mediate interactions with other proteins (Fig. 1b). *UTX/KDM6A* has been identified as a cancer driver gene via TCGA, as its loss or inactivation promotes several malignancies. JMJD3/KDM6B plays important roles in development, tissue regeneration, stem cell biology, inflammation, cellular senescence, and aging. The role of JMJD3 in cancer is poorly understood. Despite sequence similarity in the catalytic domains of JMJD3 and UTX, they have contrasting roles in various cancers, particularly leukemia.

### UTX/KDM6A as a tumor suppressor

Genomic analyses of several tumors revealed that loss of UTX occurs in various cancers, including B-cell lymphoma, bladder urothelial carcinoma, head and neck squamous cell carcinoma, pancreatic adenocarcinoma, lung squamous cell carcinoma, and kidney renal papillary cell carcinoma (Table 1)^88–93^. However, the molecular mechanism through which UTX suppresses tumor progression remains unclear. Depending on the cancer type, UTX is involved in tumor suppression, not only via its H3K27me3 demethylase activity but also via interactions with other epigenetic complexes.

Due to its H3K27me3, me2, and me1 demethylase activities, loss of UTX increases the H3K27me3 level at its target genes. Several studies showed that loss of UTX represses a certain subset of genes by increasing the levels of the repressive H3K27me3 mark, ultimately promoting proliferation, clonogenicity, adhesion, and tumorigenicity in myeloid, bladder, and lung transformation^94–97^. Upon loss of the UTX demethylases, H3K27 methyl transferases, such as PRC2/EZH2, determine the levels of H3K27me3 on the UTX-EZH2 target genes. Consistently, loss of UTX increases cellular sensitivity to EZH2 inhibition. In *UTX*-mutated multiple myeloma cells, such altered sensitivity was related to changes in gene expression triggered by a rebalancing of the H3K27me3 levels at specific genes, such as *IRF4* and *c-MYC*^94^. In *UTX/KDM6A*-mutated urothelial bladder carcinoma, PRC2/EZH2 target genes, such as *IGFBP3*, are deregulated due to H3K27me3 enrichment. EZH2 inhibition upregulated UTX-EZH2 target genes and contributed to the EZH2-dependent growth suppression in *KDM6A*-null bladder tumors in both patient-derived and cell line xenograft models^95^. PRC2/EZH2 inhibition has emerged as a potential precision therapeutic strategy for genetically defined *UTX/KDM6A* null cancers that acts by rebalancing the H3K27me3 levels at specific genes^98–100^. EZH2 inhibition also restored the normal gene expression patterns and impaired the proliferation of tumor cells harboring mutations in an H3K4 methyltransferase, MLL3/KMT2C, or a tumor suppressor, BAP1, by rebalancing the H3K27me3 levels at MLL3/BAP1 target genes^101^. In a range of human tumor types, a cancer-associated mutational hotspot was detected in the PHD domain of MLL3, which mediates association with BAP1. Cancer cells that harbored mutations in the PHD domain of MLL3 or lacked BAP1 showed reduced recruitment of UTX/KDM6A to gene enhancers, with no reduction in the levels of the repressive H3K27me3 mark. Thus, in cancer cells with *MLL3* or *BAP1* mutations, reduction of H3K27me3 via EZH2 inhibitors can restore the balance of H3K27me3 at enhancers where UTX is not properly recruited. Thus, UTX and PRC2/EZH2 inhibition contribute to enhancer restoration by preventing the deposition of excess H3K27me3^102^. Several studies showed that PRC2/EZH2 inhibition compensates for the loss of UTX function, suggesting that the catalytic activity of UTX plays an important role in tumor suppression. However, other studies showed that in certain types of cancer, an inactive UTX paralog is required for tumor development in males concomitant with loss of UTY and that the *UTX-UTY* double-knockout cells exhibited higher proliferation than the single-knockout cells, suggesting demethylase-independent tumor suppressor functions of UTX/KDM6A and UTY^103^.

Several studies showed that UTX is essential for the establishment of the active enhancer histone marks H3K4me1 and H3K27ac in a demethylase activity-independent manner via recruitment and coupling of an H3K4 methyltransferase complex (named COMPASS) and the histone acetyl transferase p300. In embryonic stem cells, loss of UTX reduced the levels of H3K4me1/H3K27ac at enhancers and transcription. An interaction between UTX and MLL4 (a COMPASS component) enhances p300-dependent H3K27 acetylation not only via UTX-dependent recruitment of p300 but also via MLL4-mediated H3K4 monomethylation. The UTX-MLL4-p300 crosstalk coordinately establishes an active enhancer for transcription^104^. Similarly, in many cancers and other diseases, enhancer-associated chromatin-modifying components, such as UTX and members of H3K4 methyltransferase complexes, are frequently mutated, leading to enhancer malfunction. In pancreatic cancer, loss of UTX causes aberrant activation of superenhancers that regulate oncogenes (such as *Delta-Np63*, *MYC*, and *RUNX3*) to drive an aggressive subtype of squamous-like pancreatic cancer^103^. To investigate the tumor-suppressive function of UTX in vivo, Gozdecka et al. developed myeloid-specific *Utx* knockout mice, most of which (63%) developed AML^105^. To mimic human leukemia, they expressed the *AML1-ETO* fusion gene in *Utx−/−* hematopoietic stem and progenitor cells. Global genomic and proteomic analyses using an *Utx*-null mouse leukemia model revealed that UTX suppresses myeloid leukemogenesis via noncatalytic functions. Loss of UTX/KDM6A leads to only minor changes in the level H3K27me3 modification, but significant changes in the levels H3K4me1/H3K27ac modifications and alterations in the binding of pioneering enhancer transcription factors, ETS and GATA. These findings suggest that loss of UTX contributes to drive tumor progression via repositioning of histone modification enzymes.

### JMJD3, a KDM6 isoform, as a tumor suppressor

JMJD3 can be considered a tumor suppressor based on the finding that it increases the expression levels of the tumor suppressors p16^INK4a^, p14^ARF^, and p15^INK4B^ and the activities of the tumor suppressors p53 and Rb. During oncogene-induced senescence (OIS) in human fibroblasts, JMJD3 demethylates the repressive H3K27me3 mark in the *INK* locus, which contains the genes encoding p16^INK4a^, p14^ARF^, and p15^INK4B^ ^106^. Oncogenes such as *B-RAF* or *RAS* induce JMJD3 expression. JMJD3 is recruited by a long noncoding-RNA, ANRIL, to the *INK* locus^107^. Cyclin-dependent kinase inhibitors, p16^INK4a^ and p15^INK4B^, maintain the activity of the Rb protein by inhibiting CDK4/6 (which normally phosphorylates and inactivates the Rb tumor suppressor), whereas p14^ARF^ increases p53 stability by inhibiting the p53-specific E3 ligase, MDM2^108^. JMJD3 also directly regulates Rb function during cellular senescence. Specific methylation of the K810 residue of Rb facilitates its interaction with CDK4 protein kinase. JMJD3 demethylates the K810 residue of Rb, which prevents CDK4 from phosphorylating the S807 and S811 residues of Rb in senescent cells^109^. Both JMJD3 and Rb are together linked to the formation of senescence-associated heterochromatin foci (SAHF). SAHF is essential for silencing genes involved in cell cycle progression. JMJD3 and p53 mutually increase their activities. p53 increases the nuclear localization of JMJD3 via protein−protein interaction and recruits JMJD3 to its target genes, such as that encoding p21, where it demethylates the H3K27me3 repressive mark^110,111^. The demethylase activity of JMJD3 eventually increases the activities of potent tumor suppressors, such as Rb and p53, leading to cellular senescence, a process that blocks tumor initiation, thereby supporting the role of JMJD3 as a tumor suppressor.

Several studies have shown potential tumor-suppressive roles for JMJD3 in different types of cancers. In glioblastoma, JMJD3 overexpression reduced the growth of glioma stem cells by increasing the p16 level and stabilizing p53 in the nucleus^112^. JMJD3 expression correlates with the p15^INK4B^ expression level in clinical colorectal cancer samples. Low JMJD3 expression correlates with poor prognosis in patients with surgically resected colorectal cancer^113^. Loss of *JMJD3* heterozygosity at chromosome 17p13.1 increased the aggressiveness of pancreatic ductal adenocarcinoma. JMJD3 expression is lower in patient samples of hepatocellular carcinoma, lung adenocarcinoma, non-Hodgkin’s lymphoma, and hematopoietic malignancies compared with that in normal tissues^106^.

### Oncogenic function of JMJD3/KDM6B

*JMJD3* is upregulated in various cancers, such as glioblastomas, breast carcinoma, melanoma, renal cell carcinoma, Hodgkin’s lymphoma, and myelodysplastic syndrome^114–119^. Furthermore, JMJD3 induces epithelial−mesenchymal transition in clear renal cell carcinoma^120^. Thus, JMJD3 plays contradictory roles as both a tumor suppressor and an oncogene. Ezh2, a H3K27 methyltransferase that counteracts JMJD3, has been reported to function as a tumor suppressor in myeloid tumors, whereas it acts as an oncogene in other tumors^121,122^. One simple but convincing explanation for the opposite roles of JMJD3 is that the effects of JMJD3 are cancer cell type-specific. For example, the absence of JMJD3 is permissive for cell division under senescence stimuli in tumors that express senescence effectors such as p16, p53, and Rb, indicating that JMJD3 functions as a tumor suppressor. However, in tumors with dysfunctional senescence or apoptotic effectors, increased JMJD3 activity is beneficial for tumor progression.

Independent of the activation of the *INK* locus, senescence is associated with changes in lysosomal activities and secretion of inflammatory cytokines, which are collectively termed the “senescence-associated secretory phenotype (SASP)”^123–125^. The SASP stimulates inflammation, aging, proliferation, and recruitment of immune and stem cells. JMJD3 has been considered a key regulator of cytokine production, as it activates NFκB and Stat1/3, which are the main transcription factors required for the induction of inflammatory cytokine expression^126–129^. Therefore, an increase in the robustness of the SASP due to JMJD3 reactivation might trigger both cellular senescence and immune responses. Conversely, JMJD3 reactivation might facilitate the recruitment of stem cells to tumors to replace old damaged cells, leading to tumor progression. Thus, JMJD3 can be considered an oncogene as it promotes development of the SASP, which enhances tumor progression.

## Conclusion

Among the several isoforms of histone demethylases, JARID1C/KDM5C and UTX/KDM6A have been identified as cancer drivers. Several studies have consistently shown that JARID1C and UTX function as tumor suppressors in various cancers, whereas the other isoforms, JARID1A, 1B, and 1D of the KDM5 subfamily and JMJD3 of the KDM6 subfamily, function as tumor suppressors and oncogenes, depending on the cancer type. The molecular mechanisms through which JARID1C and UTX are involved in tumor suppression still remain unclear. Depending on cancer type, they suppress tumor progression either through their catalytic or noncatalytic function. The observation that the *K*_m_ values for O_2_ and α-KG and the IC_50_ value for 2-HG differ among demethylases suggests that heterogeneous microenvironments and the metabolic status of individual cancer cells within solid tumors affect the heterogeneous epigenetic landscapes among single cells in a population. The localization of histone demethylases in the genome may vary with individual cell status, and metabolic control of histone demethylases may elicit epigenetic changes in a cellular context-dependent manner, which can increase cellular heterogeneity (shown in Fig. 2). Although the catalytic activities of these histone demethylases are reversibly inhibited, the consequences of temporal impairment of their activities are irreversible, as histone demethylases are involved in regulating gene expression, genome stability, and replication. A better understanding of the molecular mechanisms through which mutations in histone demethylases promote epigenetic plasticity to drive cancer progression will guide precision therapeutic strategies for the selection of histone demethylase and methyl-transferase inhibitors.
